# Microarray profiling of hypothalamic gene expression changes in Huntington’s disease mouse models

**DOI:** 10.3389/fnins.2022.1027269

**Published:** 2022-11-03

**Authors:** Elna Dickson, Amoolya Sai Dwijesha, Natalie Andersson, Sofia Lundh, Maria Björkqvist, Åsa Petersén, Rana Soylu-Kucharz

**Affiliations:** ^1^Biomarkers in Brain Disease, Department of Experimental Medical Science, Lund University, Lund, Sweden; ^2^Translational Neuroendocrine Research Unit, Department of Experimental Medical Science, Lund University, Lund, Sweden; ^3^Pathways of Cancer Cell Evolution, Division of Clinical Genetics, Department of Laboratory Medicine, Lund University, Lund, Sweden

**Keywords:** Huntington’s disease, neuroendocrine, hypothalamus, microarray, HD mouse models, huntingtin, differential expression

## Abstract

Structural changes and neuropathology in the hypothalamus have been suggested to contribute to the non-motor manifestations of Huntington’s disease (HD), a neurodegenerative disorder caused by an expanded cytosine-adenine-guanine (CAG) repeat in the huntingtin (HTT) gene. In this study, we investigated whether hypothalamic HTT expression causes transcriptional changes. Hypothalamic RNA was isolated from two different HD mouse models and their littermate controls; BACHD mice with ubiquitous expression of full-length mutant HTT (mHTT) and wild-type mice with targeted hypothalamic overexpression of either wild-type HTT (wtHTT) or mHTT fragments. The mHTT and wtHTT groups showed the highest number of differentially expressed genes compared to the BACHD mouse model. Gene Set Enrichment Analysis (GSEA) with leading-edge analysis showed that suppressed sterol- and cholesterol metabolism were shared between hypothalamic wtHTT and mHTT overexpression. Most distinctive for mHTT overexpression was the suppression of neuroendocrine networks, in which qRT-PCR validation confirmed significant downregulation of neuropeptides with roles in feeding behavior; hypocretin neuropeptide precursor (*Hcrt*), tachykinin receptor 3 (*Tacr3*), cocaine and amphetamine-regulated transcript (*Cart*) and catecholamine-related biological processes; dopa decarboxylase (*Ddc*), histidine decarboxylase (*Hdc*), tyrosine hydroxylase (*Th*), and vasoactive intestinal peptide (*Vip*). In BACHD mice, few hypothalamic genes were differentially expressed compared to age-matched WT controls. However, GSEA indicated an enrichment of inflammatory- and gonadotropin-related processes at 10 months. In conclusion, we show that both wtHTT and mHTT overexpression change hypothalamic transcriptome profile, specifically mHTT, altering neuroendocrine circuits. In contrast, the ubiquitous expression of full-length mHTT in the BACHD hypothalamus moderately affects the transcriptomic profile.

## Background

Huntington’s disease (HD) is a fatal neurodegenerative disorder caused by a CAG repeat expansion in exon one in the huntingtin (*HTT*) gene (The Huntington’s Disease Collaborative Research Group, 1993). The expanded repeat results in the formation of a mutant HTT protein (mHTT) with an abnormally long polyglutamine (polyQ) stretch, associated with protein misfolding and aggregation of the mutant protein in neurons (The Huntington’s Disease Collaborative Research Group, 1993; Bates et al., 2015). The length of CAG repeats is inversely correlated to the age of onset, with 40 or more CAG repeats resulting in full-penetrance and 60 or more repeats to juvenile HD (Rubinsztein et al., 1996; Brinkman et al., 1997). Currently, there is no cure for HD. Gene-silencing approaches are tested in clinical trials, including interventions that either selectively silence the mutant *HTT* or both mutant and normal alleles (Wild and Tabrizi, 2017; Tabrizi et al., 2019), which stresses the need for further understanding of normal HTT function in cells. Several *in vitro* and *in vivo* studies showed that normal HTT is involved in numerous cellular processes, including cell maturation, vesicle trafficking, synaptic transmission, and neuroprotection [reviewed in Cattaneo et al. (2005), van der Burg et al. (2009), Saudou and Humbert (2016), and Barron et al. (2021)].

The hypothalamus plays a primary role in the central-peripheral regulatory network that maintains body homeostasis, including regulating whole-body energy metabolism (Anand and Brobeck, 1951; Brown et al., 2012; Waterson and Horvath, 2015; Feldman et al., 2016). Metabolic alterations and other non-motor symptoms with a hypothalamic link are present throughout all stages of HD and have been shown in HD animal models (Kirkwood et al., 2001; Djoussé et al., 2002; Duff et al., 2007; Mochel et al., 2007; Hult et al., 2011; Hult Lundh et al., 2013; Dickson et al., 2022). Imaging and post-mortem studies in clinical and animal models revealed pathological changes such as atrophy, reduced gray matter content, and loss of neuropeptides in the hypothalamus, some of that can be detected even before the onset of motor features (Kassubek et al., 2004; Petersén et al., 2004; Kotliarova et al., 2005; Aziz et al., 2008; Politis et al., 2008; Soneson et al., 2010). In addition, a higher baseline body mass index (BMI) is associated with slower disease progression (van der Burg et al., 2017). However, prior studies on high caloric diets and the use of transgenic mice to induce weight gain appear insufficient to significantly modify disease features in HD animal models (Sanberg et al., 1981; Fain et al., 2001; Trejo et al., 2004; van der Burg et al., 2008; Marder et al., 2009; Sjögren et al., 2019). In line with this, van der Burg et al. followed up on results from the original BMI and disease progression study (van der Burg et al., 2017) and reported no causal relation between BMI and age of onset in clinical HD (van der Burg et al., 2021). Further studies are required on the normal HTT and mHTT functions in crucial areas for metabolic control, including the hypothalamus, to elucidate mechanisms underlying metabolic changes in HD.

Transcriptional dysregulation in the striatum is one of the hallmarks of HD (Zucker et al., 2005; Thomas, 2006; Malaiya et al., 2021). Specific gene expression changes using qRT-PCR have also been reported in the HD hypothalamus (Hult et al., 2011; Soylu-Kucharz et al., 2015; Baldo et al., 2019). Large-scale transcriptome analysis has not been performed to identify the hypothalamic alterations after targeted wtHTT and mHTT expression. Therefore, in this study, we performed microarray profiling of hypothalamic samples from two different HD mouse models; the transgenic BACHD mice [bacterial artificial chromosome (BAC)-mediated; full-length mHTT] and wild-type mice with hypothalamus targeted overexpression of N-terminal HTT fragments (wtHTT 18Q; AAV-HTT853-18Q or mHTT 79Q; AAV-HTT853-79Q). Both models share the feature of increased food intake and early weight gain but differ in the rate of disease progression and extent of hypothalamic pathology (Gray et al., 2008; Hult et al., 2011). We analyzed each mouse model using their age-matched wild-type (WT) littermates as control groups. The AAV datasets wtHTT 18Q vs. WT and mHTT 79Q vs. WT had the highest number of significantly altered genes across models. Gene Set Enrichment Analysis (GSEA) identified a set of shared genes between wtHTT 18Q vs. WT and mHTT 79Q vs. WT related to sterol- and cholesterol processes. Further, gene sets associated with suppressed feeding behavior were among the top-ranked KEGG pathways in the mHTT 79Q vs. WT dataset. Taken together, our data support transcriptional dysregulation as an essential mechanism of action for mHTT in inducing hypothalamic pathology in HD animal models.

## Materials and methods

### Ethical considerations

All the mice used in the study were housed in groups and maintained at a 12 h light/dark cycle with free access to a standard chow diet and water. All the experimental procedures performed on mice were carried out in accordance with the approved guidelines in the ethical permits approved by Lund University Animal Welfare and Ethics committee in the Lund-Malmö region (Ethical permit numbers: 12585/2017, M20-11, M65-13, and M135-14).

### Animals

Microarray profiling of the hypothalamic transcriptome was performed in Adeno-associated viral (AAV) vector-mediated groups of WT mice with targeted expression of wtHTT (AAV-HTT853-18Q) or mHTT (AAV-HTT853-79Q) fragments and BACHD mice that express full-length mHTT (97Q) (Gray et al., 2008). Both HD models were compared to age-matched WT controls. All mice used in the study were females from the FVB/N strain.

AAV vector-mediated HD models achieve region-specific overexpression of HTT fragments in the brain through targeted injections using stereotactic surgery. AAV groups were assessed at 4 weeks post-injection since this was the earliest timepoint for a significant weight gain, as shown in previous studies (Hult et al., 2011; Dickson et al., 2022). The vector constructs used in the present study was a recombinant AAV vector of serotype 5 (rAAV5) carrying an 853 amino acid N-terminal HTT fragment corresponding to either wtHTT (18 CAG repeats; AAV-HTT853-18Q) or mHTT (79 CAG repeats; AAV-HTT853-79Q) under control by the human Synapsin-1 (Syn-1) promoter (de Almeida et al., 2002). Stereotactic injections in the hypothalamus were performed as described previously (Hult et al., 2011). In brief, 8 weeks old WT female mice were bilaterally injected, and the surgeries were performed under isoflurane anesthesia. The anterior-posterior (AP) and medial-lateral (ML) stereotaxic coordinates for the hypothalamus were determined according to bregma, and dorsal-ventral (DV) coordinates were calculated from the dura mater. The hypothalamic coordinates were AP = 0.6 mm, ML = 0.6 mm, and DV = 5.3 mm. A total viral vector volume of 0.5 μl was delivered in each hemisphere. Following an initial injection of 0.1 μl of viral vector solution, 0.05 μl of viral vectors were delivered in intervals of 15 s. Following the injection, the glass capillary was left in the target for an additional 5 min. The vectors and titers were as follows: rAAV5-hSyn-HTT853-18Q: 1.3E + 14 GC/ml, and rAAV5-hSyn-HTT853-79Q: 1.2Ex14 GC/ml. A group of WT littermates (uninjected mice) was kept as a control group. Group numbers for each AAV group were: 18Q: *n* = 5, 79Q: *n* = 8 and WT control: *n* = 5.

Bacterial artificial chromosome-mediated transgenic mouse model is a transgenic mouse model of HD and ubiquitously expresses a full-length human mHTT (97 CAG repeats; 97Q) (Gray et al., 2008). In the study, BACHD mice were assessed at two time points, one group representing the early (2 months of age) and the second group late stages (10 months of age) of the disease in comparison to their WT littermate controls (Gray et al., 2008; Hult et al., 2011). For 2 months of age, group numbers were the following: BACHD: *n* = 6 and WT: *n* = 6, and for 10 months of age: BACHD: *n* = 5 and WT: *n* = 3.

### Tissue collection and RNA extraction

Hypothalamic tissue was dissected on ice and snap-frozen in liquid nitrogen after a terminal dose of sodium pentobarbital (600 mg/kg, Apoteksbolaget, Lund, Sweden) via intraperitoneal injection. Total RNA was extracted using the RNeasy Lipid Tissue Mini Kit (Qiagen Inc, Hilden, Germany) according to the manufacturer’s instructions. RNA concentration and RNA quality measured in terms of RNA integrity number (RIN) were determined using the Agilent 2,100 Bioanalyzer (Agilent Technologies, Santa Clara, CA, USA). Samples with poor RIN (<7) were omitted from further analysis. Microarray analysis was performed on total hypothalamic RNA using the Affymetrix platform (Mouse Gene ST 1.0 array; Affymetrix platform - Thermo Fisher Scientific, Santa Clara, CA, USA).

### Data pre-processing and limma

Analyses were made using R v.4.1.1 (R Core Team, 2022). Raw .CEL-files obtained from the microarray analysis using the Affymetrix platform (34,760 variables) were imported using ReadAffy, followed by pre-processing of the raw data set using Robust Multi-Array Averaging (RMA); both functions were from the affy package (v. 1.70.0) (Gautier et al., 2004). A design matrix was created to illustrate analysis groups for the samples. WT samples were used as a reference level. A linear model was fitted for each gene using lmFit. MakeContrasts was used to specify which groups to compare, followed by contrasts.fit to perform the comparison (Phipson et al., 2016). Subsequently, empirical Bayes smoothing was applied to the standard errors (limma, v.3.48.3) (Ritchie et al., 2015). This resulted in a result matrix for each contrast comparison. For downstream analyses, probe IDs with “NA” were excluded. Outputs from limma can be found in Supplementary Data Sheet 1.

### Heatmaps

Limma output files for 18Q vs. WT and 79Q vs. WT were sorted based on adj. *p*-value followed by sorting it from the highest log2 (FC) to the lowest. The top 10 most upregulated and top 10 most downregulated significantly differentially expressed genes were identified, and their respective RMA-limma values were used to construct and color the heatmap. Heatmaps were generated using the pheatmap package in R^1^ (v. 1.0.12) (Warnes et al., 2015). Hierarchical clustering was performed using Euclidean distance and average linkage. RMA-limma values were scaled using the function scale().

### Functional Annotation Clustering in database for annotation, visualization, and integrated discovery

Gene lists of 18Q vs. WT and 79Q vs. WT used for DAVID Functional Annotation Clustering were retrieved by filtering each limma dataset to only retrieve genes with an adj. *p*-value < 0.05. The 18Q vs. WT and 79Q vs. WT datasets were subsequently sorted into three gene sets: shared genes, unique genes for 18Q vs. WT, and unique genes for 79Q vs. WT. The shared and unique gene lists (ENTREZ IDs) were imported into DAVID^2^ (Huang da et al., 2009; Sherman et al., 2022) for Functional Annotation Clustering. Three categories were used: UP_KW_BP, GOTERM_BP_DIRECT, and KEGG_PATHWAY. The default settings for Functional Annotation Clustering were used, including the Classification Stringency of “Medium.” Gene lists with Annotation Summary Results and Functional Annotation Clustering outputs from DAVID are listed in Supplementary Data Sheet 2.

### Gene set enrichment analysis

ClusterProfiler (v.4.0.5) was used to perform GSEA (Yu et al., 2012; Wu et al., 2021). The function gseGO was used to assess the enrichment of GO terms and gseKEGG to assess enrichment of KEGG pathways. The analysis was performed on the entire gene list obtained from the limma analysis, besides the removal of probe IDs to which no gene could be mapped (“NA”). As organism, mogene10sttranscriptcluster.db was used, and mmu for KEGG (Kanehisa et al., 2016; MacDonald, 2017) (KEGG^3^^4^). The reported *p*-values were further adjusted using the Benjamini-Hochberg procedure to correct for multiple testing. Outputs from GSEA can be found in Supplementary Data Sheet 3 (GO) and Supplementary Data Sheet 4 (KEGG).

### Quantitative real-time polymerase chain reaction validation of microarray data

To synthesize cDNA, 1 μg of RNA from each sample was reverse transcribed using SuperScript IV Reverse Transcriptase SuperScript IV kit (Invitrogen, Carlsbad, CA, USA) according to the manufacturer’s instructions. Mouse qRT-PCR primers were designed using Primer3Plus software (Untergasser et al., 2012). qRT-PCR reactions were carried out in triplicates following a three-step amplification protocol using the LightCycler 480 system (Roche, Basel, Switzerland). The ΔΔCT method (Livak and Schmittgen, 2001) was used to calculate gene expression changes relative to housekeeping genes β-actin and glyceraldehyde 3-phosphate dehydrogenase (GAPDH). Primer sequences are listed in Supplementary Table 1.

### Statistical analyses

Statistical analysis of qRT-PCR data was performed using Graphpad Prism 9 (Version 9.4.1, San Diego, CA USA). (GraphPad software - San Diego, CA USA). Data were analyzed using non-parametric Mann-Whitney U tests with a *p*-value < 0.05 considered statistically significant.

## Results

### Differential expression analysis of microarray profiling data from adeno-associated viruses and bacterial artificial chromosome-mediated transgenic mouse models using linear models for microarray data analysis

**FIGURE 1:**
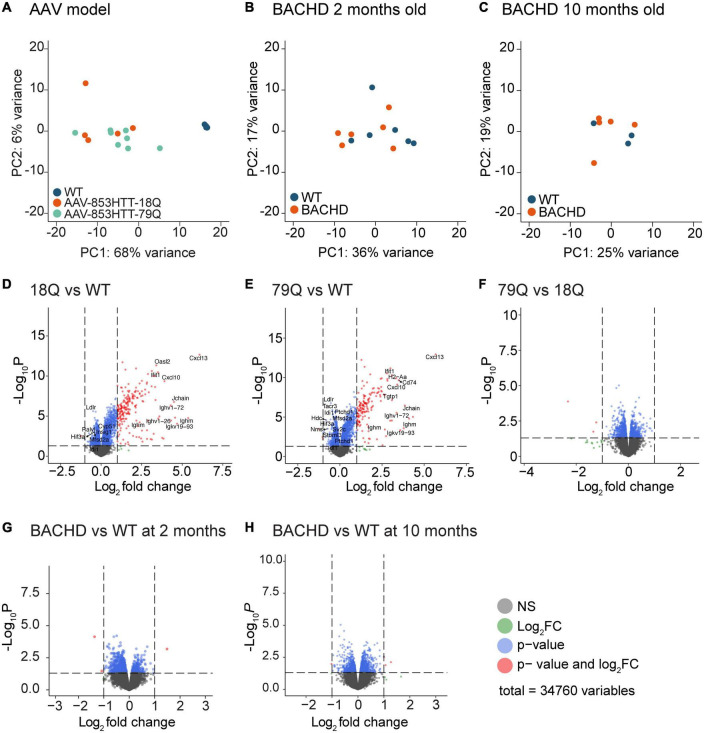
Limma of microarray datasets show the most pronounced effect on gene expression in mouse groups overexpressing huntingtin (HTT) in the hypothalamus. A total of 34,760 variables (probe IDs) from the Affymetrix platform were analyzed by limma. **(A)** Principal Component Analysis (PCA) of all samples from the AAV groups was analyzed at 4 weeks post-injection. **(B)** PCA of samples from bacterial artificial chromosome (BAC)-mediated transgenic mouse model (BACHD) mice compared to age-matched controls at 2 months of age. **(C)** PCA of samples from BACHD mice compared to age-matched controls at 10 months of age. **(D–F)** Volcano plots for the AAV groups. Color key for variables: gray = non-significant (*p*-value > 0.05), blue = *p*-value < 0.05, and red = *p*-value < 0.05 and log2(FC) > 1 or log2(FC) < −1. Gene names of the top 10 upregulated and top 10 down-regulated genes when filtered for adj. *p*-value and log2(FC) are displayed. For the 79Q vs. 18Q dataset, no gene passed adj. *p*-value < 0.05, instead the top genes are marked based on only log2(FC) (Supplementary Data Sheet 1). **(G,H)** Volcano plots for the BACHD groups; 2 months of age (early stage of disease) and 10 months of age (late stage) (Supplementary Data Sheet 1). No gene passed adj. *p*-value < 0.05; displayed are the top 10 upregulated and top 10-downregulated genes based on only log2(FC). AAV, adeno-associated viral vector; 79Q, HTT853-79Q vector; 18Q, HTT853-18Q vector; Limma, linear models for microarray data.

Principal component analysis (PCA) of the microarray data showed a clear separation of the AAV-HTT groups from the WT samples (Figure 1A). PCA of BACHD mice at 2 and 10 months showed no clear separation compared to WT (Figures 1B,C). Volcano plots of log2 (FC) and the *p*-value in the limma datasets comparing injected mice to uninjected (18Q vs. WT and 79Q vs. WT) showed a skewed distribution with a preference for upregulated genes in the injected mice (Figures 1D,E). Filtering the dataset based on Benjamini-Hochberg adjusted *p*-values < 0.05 resulted in 735 variables in 18Q vs. WT and 721 in 79Q vs. WT. In the 79Q vs. 18Q dataset that compares the HTT vector injected groups, none of the variables passed the adj. *p* < 0.05 cutoff (Figure 1F).

With a *p* < 0.05 cutoff, 1,422 variables remained for BACHD 2 months vs. WT (Figure 1G) and 1,648 variables for BACHD 10 months vs. WT (Figure 1H), but none of the variables passed filtering for adj. *p* < 0.05.

### A set of significantly altered genes related to sterol- and cholesterol processes are shared between mice overexpressing wild-type and mutant huntingtin fragments

**FIGURE 2:**
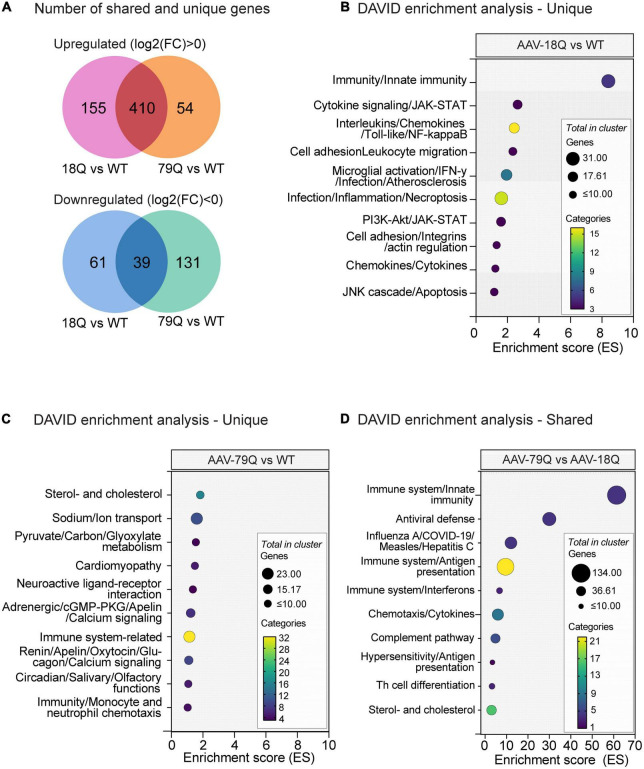
DAVID Functional Annotation Clustering of unique and shared genes between the 18Q vs. WT and 79Q vs. Wild type (WT) datasets. 18Q vs. WT and 79Q vs. WT datasets were filtered based on adj. *p* < 0.05 and separated into three gene sets: shared genes, genes unique for 18Q vs. WT, and genes unique for 79Q vs. WT. Functional Annotation Clustering was performed using the DAVID bioinformatics resource (Huang da et al., 2009; Sherman et al., 2022) on each gene set. Categories used for clustering were KEGG pathways and Biological process (BP) from GO and keyword BP from Uniprot. **(A)** Venn diagram showing the number of shared and unique genes between 18Q vs. WT and 79Q vs. WT, shown as separated by log2(FC) < 0 and log2(FC) > 0. **(B–D)** The top 10 DAVID annotation clusters (based on enrichment score, ES). The y-axis denotes annotation clusters; the full output and lists of each respective gene set can be found in Supplementary Data Sheet 2. **(B)** Unique gene set (adj. *p* < 0.05) in 18Q vs. WT, **(C)** unique gene set (adj. *p* < 0.05) in 79Q vs. WT, and **(D)** shared gene set (adj. *p* < 0.05) between the 18Q vs. WT and 79Q vs. WT datasets. DAVID, database for annotation, visualization, and integrated discovery; GO, gene ontology; ES, enrichment score; 79Q, HTT853-79Q vector; 18Q, HTT853-18Q vector; limma, linear models for microarray data, FC, fold change.

The 18Q vs. WT and 79Q vs. WT datasets had the highest number of significant (adj. *p* < 0.05) variables, and we further analyzed the data using the Database for Annotation, Visualization and Integrated Discovery (DAVID) (see text footnote 2) (Huang da et al., 2009; Sherman et al., 2022). Previous studies have shown that wtHTT and mHTT impact the body to varying degrees [reviewed in Cattaneo et al. (2005), van der Burg et al. (2009), and Saudou and Humbert (2016)], highlighting the effects of both gain and loss of HTT functions. However, increased body weight can be caused by both wtHTT and mHTT expression in mice, where the most notable increase is displayed by mice expressing mHTT (Van Raamsdonk et al., 2006; Hult et al., 2011; Baldo et al., 2013; Dickson et al., 2022). Furthermore, HD mouse models expressing full-length HTT (i.e., BACHD, YAC18) also share a similar phenotype (Pouladi et al., 2010; Hult et al., 2011). Therefore, we compared significant genes (adj. *p* < 0.05) identified by limma between the 18Q vs. WT and 79Q vs. WT datasets (Supplementary Data Sheet 1), using the DAVID tool to investigate the functional annotation of shared and unique genes between the HTT groups (18Q vs. WT and 79Q vs. WT) (Figure 2A). DAVID Functional Annotation Clustering was performed using three categories: Biological Process (BP) from gene ontology (GO), BP keywords from Uniprot, and KEGG pathways.

For 18Q vs. WT, the unique gene set consisted of 216 genes, 155 with log2(FC) > 0 and 61 with log2(FC) < 0 (Figure 2A). Functional Annotation Clustering of the 18Q vs. WT unique gene set showed clusters related to inflammation and the immune system in the top 10 [enrichment score (ES) range = 8.40–1.16] (Figure 2B). Comparing log2(FC) of genes in the top cluster in 18Q vs. WT [*n* = 51 genes, (adj. *p* < 0.05) to 79Q vs. WT (adj. *p* > 0.05)] showed that the gene with the highest difference in log2(FC) was immunoglobulin heavy chain (X24 family) (*Igh-VX24)* followed by tripartite motif-containing 30D (*Trim30d*) and toll-like-receptor 7 (*Tlr7*) [log2(FC): (18Q vs. WT–79Q vs. WT); *Igh-Vx24*: 1.04, *Trim30d*: 0.42, *Tlr7*: 0.34]. Across all 51 genes, the mean difference with 95% CI was 0.135 (0.090, 0.181) (Supplementary Data Sheet 1, 2).

For 79Q vs. WT, the unique gene set consisted of 185 genes, 54 with log2(FC) > 0 and 131 with log2(FC) < 0 (Figure 2A). Among the top 10 annotation clusters for 79Q vs. WT were sterol- and cholesterol-related terms, neuropeptide function, and immune-related pathways (ES range = 1.83–1.03) (Figure 2C). Comparison of the log2(FC) of genes in the top cluster (sterol- and cholesterol-related) in 79Q vs. WT (*n* = 12 genes, adj. *p* < 0.05) to 18Q vs. WT (adj. *p* > 0.05) showed a mean difference with 95% CI of -0.042 (-0.078, -0.006). In the top 5/10 cluster related to neuroactive ligands (GO:0032355∼response to estradiol, GO:0007568∼aging, mmu04080∼Neuroactive ligand-receptor interaction, *n* = 12 genes), we found a mean difference in log2(FC) of -0.067 (-0.141, 0.006) between the 79Q vs. WT and 18Q vs. WT datasets, where the genes with the highest difference were proenkephalin (*Penk*) and tachykinin receptor 3 (*Tacr3*) [log2(FC): (79Q vs. WT-18Q vs. WT); *Penk*: -0.26, *Tacr3*: -0.24] (Supplementary Data Sheets 1, 2).

The shared gene list between 18Q vs. WT and 79Q vs. WT consisted of 410 genes with log2(FC) > 0 and 39 genes with log2(FC) < 0 (Figure 2D and Supplementary Data Sheet 2). An immune system-related annotation cluster was top-ranked (ES = 61.36), followed by enrichment of clusters involving immune system components and diseases related to immunity (Figure 4D). Sterol-and cholesterol-related terms constituted annotation cluster 10 (ES = 2.99) containing 37 genes such as low-density lipoprotein receptor [*Ldlr*; log2(FC) 18Q vs. WT: −0.49, 79Q vs. WT: −0.54] (Supplementary Data Sheets 1, 2).

**FIGURE 3:**
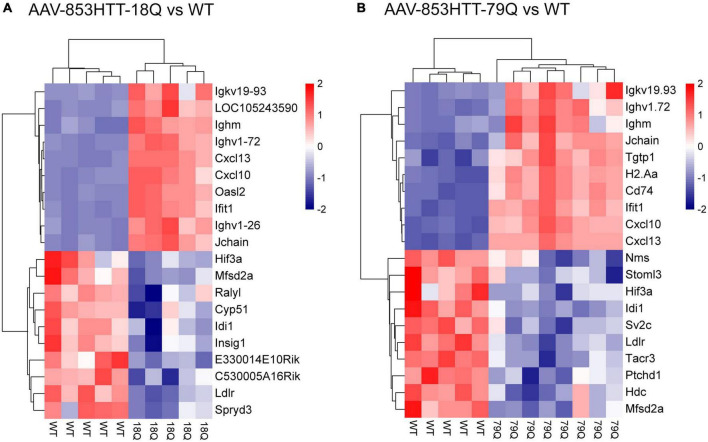
Top-ranked downregulated genes in the 79Q vs. wild type (WT) dataset include histidine decarboxylase (*Hdc*), tachykinin receptor 3 (*Tacr3*), and neuromedin S (*Nms*). Heatmap of scaled RMA-limma values [scale(rows)] with hierarchical clustering showing the top 10 up- and top 10 downregulated genes, based on adj. *p* and log2(FC) in **(A)** 18Q vs. WT and **(B)** 79Q vs. WT. *Hdc*, *Tacr, Nms*, and *Sv2c*, associated with neuroendocrine signaling, were significantly altered (criteria adj. *p* < 0.05) in the 79Q vs. WT limma dataset. 79Q, HTT853-79Q vector; 18Q, HTT853-18Q vector; Sv2c, synaptic vesicle glycoprotein 2C.

**FIGURE 4:**
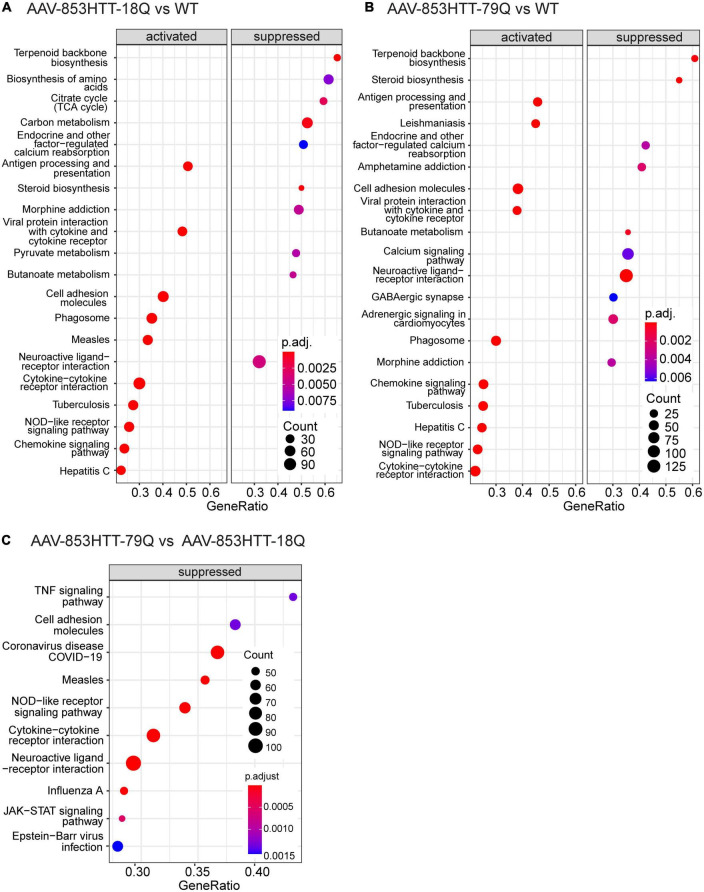
Gene set enrichment analysis (GSEA) of KEGG pathways comparing mutant huntingtin (mHTT) 79Q to wild-type huntingtin (wtHTT) 18Q indicate suppressed pathways related to the immune system and neuroendocrine signaling. GSEA of KEGG pathways was performed on the limma output files for the AAV datasets (79Q vs. 18Q, 18Q vs. WT and 79Q vs. WT). **(A)** The top 10 up- and top 10 downregulated KEGG pathways in AAV-853HTT-18Q vs. WT and **(B)** AAV-853HTT-79Q vs. WT. **(C)** For AAV-853HTT-79Q vs. AAV-853HTT-18Q, all pathways identified by GSEA-KEGG had a negative normalized enrichment score (NES), indicating suppression. The top 10 are shown. AAV, adeno-associated viral vector; 79Q, HTT853-79Q vector; 18Q, HTT853-18Q vector.

### Top-ranked downregulated genes in mice overexpressing huntingtin show functional associations with biosynthetic- and neuroendocrine processes

Next, we assessed the top 10 up- and top 10 downregulated genes [adj. *p* and log2(FC)] in the 18Q vs. WT and 79Q vs. WT datasets (Supplementary Data Sheet 1). For 18Q vs. WT, among the top 10 downregulated genes were *Ldlr*, isopentenyl-diphosphate delta isomerase 1 (*Idi1*), and major facilitator superfamily domain-containing 2A (*Mfsd2a*) (Figure 3A), genes that were also among top 10 downregulated in the 79Q vs. WT dataset. In addition, part of the top 10 downregulated genes in 79Q vs. WT were neuromedin S (*Nms*), Hdc, synaptic vesicle glycoprotein 2C (*Sv2c*), and *Tacr3* (Figure 3B). Comparing the top 10 upregulated genes between 18Q vs. WT and 79Q vs. WT showed that seven out of 10 were shared and related to the immune system.

Next, we elaborated further on the functional implications of the differences in the top 10 downregulated genes between 18Q vs. WT and 79Q vs. WT. GSEA of GO-BP with leading-edge analysis (Subramanian et al., 2005) was used to consider the cross-correlation of the top 10 downregulated genes with changes across the whole limma dataset and relation to biological processes (Supplementary Data Sheet 3). The top 10 downregulated genes were compared to leading-edge gene sets in the GSEA-GO BP outputs. For the top five GSEA-GO BP (ranked by normalized enrichment score: NES), the top 10 downregulated genes were related to biosynthesis and/or metabolism of sterol, cholesterol, isopentenyl diphosphate, and phosphatidylcholine. For 79Q vs. WT, multiple of the top 10 downregulated genes could be attenuated in leading-edge subsets (top five GSEA-GO BP per gene) for behavioral processes (“behavior,” “circadian behavior,” “feeding behavior”) and neuropeptide signaling pathways. Taken together, at 4 weeks post-injection, wtHTT and mHTT overexpression groups share genes that are involved in biosynthetic pathways. However, specific to mHTT overexpression are significant decreases in key neuropeptides, an effect that is not found or of a lower magnitude for wtHTT overexpression.

### Hypothalamic wild-type and mutant huntingtin overexpression causes widespread alteration of biosynthetic pathways

Next, we used GSEA with a leading-edge analysis of KEGG pathways (Subramanian et al., 2005). As shown in Figure 1, none of the genes passed adj. *p*-value < 0.05 for the 79Q vs. 18Q and BACHD datasets. By using GSEA, we may still identify biologically relevant pathways that exhibit noteworthy cross-correlation between genes with a subtle change in expression levels or weak statistical significance (Supplementary Data Sheet 4).

GSEA-KEGG of 18Q vs. WT and 79Q vs. WT datasets showed that the majority of the significantly enriched KEGG pathways in the top-ranked list (the top 10 NES > 0 and top 10 NES < 0: Figures 4A,B) were shared and related to biosynthesis (“Terpenoid backbone synthesis,” “Steroid biosynthesis”), inflammation and immune system (“Antigen processing and presentation,” “Cytokine-cytokine receptor interaction”), and neuroendocrine signaling (“Endocrine and other factor-regulated calcium reabsorption,” “Neuroactive ligand-receptor interaction”).

In the 79Q vs. 18Q dataset, all significantly enriched pathways identified by GSEA (*n* = 38, Supplementary Data Sheet 4) had a negative NES score, indicating suppression. The top 10 pathways were related to the immune system and “Neuroactive ligand-receptor interaction” (Figure 4C). For “Neuroactive ligand-receptor interaction” (mmu04080, NES = −1.93) the mean with 95% CI of the leading-edge gene set (*n* = 108, adj. *p* > 0.05) was -0.156 (-0.192, -0.121). The leading- edge gene with the lowest log2(FC) was prolactin (*Prl*) at -1.63 followed by growth hormone (*Gh*) -1.02, Vip -0.59, the alpha subunit of glycoprotein hormones (*Cga*) -0.53 and hypocretin (*Hcrt*) -0.35 (Supplementary Data Sheets 1, 4).

For 2 months old BACHD vs. WT, GSEA-KEGG identified three pathways; “Glutamatergic synapse” (mmu04724, NES = 1.69), “Biosynthesis of unsaturated fatty acids” (mmu01040, NES = −2.03) and “Fatty acid elongation” (mmu00062, NES = −2.06) (Supplementary Image 1A). Among leading-edge genes that had high fold change vs. WT and difference in the 10 months old BACHD vs. WT dataset were acyl-CoA thioesterase 5 (*Acot5*) and vesicular glutamate transporter 1 (*Slc17a7*) that were respectively part of the fatty-acid related and “Glutamatergic synapse” pathways [log2(FC); *Acot5*: 2 months vs. WT: 0.33, 10 months vs. WT: -0.09; *Slc17a7*: 2 months vs. WT: -0.46, 10 months vs. WT: 0.43]. None of the three pathways identified in 2 months old BACHD vs. WT were significant in GSEA-KEGG for 10 months old BACHD vs. WT. When comparing each leading-edge gene set in 2 months old BACHD vs. WT to the same genes in 10 months BACHD vs. WT, the mean log2(FC) difference was <0.2 for all three pathways. Instead, for 10 months old BACHD vs. WT, fourteen KEGG pathways were identified (Supplementary Image 1B). Immune-related pathways such as “Antigen processing and presentation” (mmu04612, NES = 1.90) had the highest number of leading-edge gene overlap (Supplementary Data Sheet 4). Histocompatibility 2, class II antigen A, alpha (*H2-Aa*), and *Cd74* were among the leading-edge genes with the highest log2(FC) and difference from the 2 months of age vs. WT dataset [log2(FC); *H2-Aa*: 10 months vs. WT: 0.73, 2 months vs. WT: 0.09 and *Cd74*: 10 months vs. WT: 0.99, 2 months vs. vs. WT: 0.05]. Pathways related to gonadotropin-releasing hormone (*Gnrh*), all NES > 0, were also found in 10 months old BACHD vs. WT, where the alpha subunit of glycoprotein hormones (*Cga*) was among the leading-edge genes with the highest fold change [log2(FC); *Cga:* 10 months vs. WT: 1.66, 2 months vs. WT: -0.35]. Only one of the 14 KEGG pathways identified by GSEA had a negative NES of -1.95, indicating suppression; “Oxidative phosphorylation” (mmu00190). The overall change of the 59 genes in the leading-edge gene set was -0.056 (-0.067, -0.045) [mean log2(FC) with 95% confidence interval (95% CI)].

### Quantitative real-time polymerase chain reaction confirms selective mutant huntingtin-mediated loss of key enzymes in dopamine- and histamine synthesis in the hypothalamus of mice overexpressing mutant huntingtin

**FIGURE 5:**
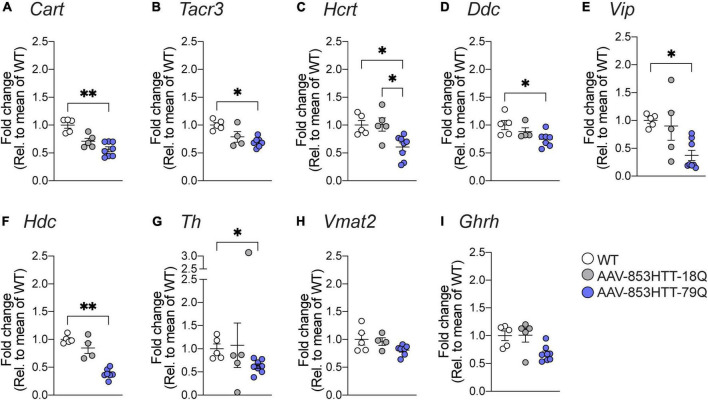
Quantitative real-time polymerase chain reaction (qRT-PCR) validation of differentially expressed genes in wtHTT 18Q and mHTT 79Q groups. Gene expression analysis of candidate genes was performed with hypothalamic RNA samples from mice with targeted mutant HTT (79Q) overexpression and wild-type HTT (18Q) overexpression (4 weeks post-injection). Data are expressed as mRNA expression relative to the mean of uninjected WT controls. **(A)** Cocaine and amphetamine-regulated transcript (*Cart*, *p* = 0.0031), **(B)** Tachykinin receptor 3 (*Tacr3, p* = 0.0148), **(C)** Hypocretin neuropeptide precursor (*Hcrt*, WT vs. 79Q: *p* = 0.0326, 18Q vs. 79Q: *p* = 0.0393), **(D)** Dopa decarboxylase (*Ddc, p* = 0.0136), **(E)** Vasoactive intestinal peptide (*Vip, p* = 0.0149), **(F)** Histidine decarboxylase (*Hdc, p* = 0.0032), **(G)** Tyrosine hydroxylase (*Th*, p = 0.0257), **(H)** Vesicular monoamine transporter 2 (Vmat2; encoded by *Slc18a2*), and **(I)** Growth hormone-releasing hormone (*Ghrh*). The data are represented as scatter dot plots, and bars represent mean ± SEM, and the Kruskal-Wallis test, followed by Dunn’s multiple comparisons, was used to compare the groups. *Denotes *p*-value is 0.01 to 0.05 and **denotes *p*-value is 0.001 to 0.01.

Using qRT-PCR, we validated a set of candidate genes. As described above, for 79Q vs. WT examined at 4 weeks post-injection, we found transcriptional downregulation in GO-terms and pathways related to the neuroendocrine system, in particular for feeding responses. The leading-edge gene set for “Feeding behavior” (GO:008631) that was among the top suppressed processes in GSEA-GO BP consisted of 57 genes (Supplementary Data Sheet 3). We have previously shown significant downregulation of hypocretin neuropeptide precursor (*Hcrt*) in mice with mHTT 79Q overexpression compared to wtHTT 18Q groups and uninjected WT controls (Soylu-Kucharz et al., 2015; Baldo et al., 2019). Here, in addition to *Hcrt*, we quantified mRNA levels of other neuropeptides. At the 4 weeks post-injection, *Cart*, *Tacr3*, and *Hcrt* gene expressions were significantly downregulated in the 79Q group compared to the WT control, while no significant differences were found for the 18Q group in the same comparison (Figures 5A–C).

We also observed associations to the catecholamine system that were most prominent in 79Q, such as *Hdc* as part of the top 10 downregulated genes and related leading-edge subsets in GSEA. Comparing GSEA-GO BP outputs between the 79Q vs. WT and 18Q vs. WT datasets showed a higher number of catecholamine-related processes that were only significant for the 79Q vs. WT dataset (Supplementary Data Sheet 3). We therefore expanded the qRT-PCR analysis to include critical genes involved in the catecholamine system. The 79Q group displayed significant downregulations in *Ddc*, *Vip, Hdc*, and *Th* compared to the WT control (Figures 5D–G). No significant difference was found for the vesicular monoamine transporter 2 (*Vmat2*, *Slc18a2*) or Growth hormone-releasing hormone (*Ghrh*) (Figures 5H,I). A match of validated genes with leading-edge subsets in GSEA-GO BP showed that *Ddc*, *Th*, *Tacr3*, and *Hdc* were part of catecholamine and catechol-containing metabolic processes while *Vip* and *Cartpt* in catecholamine secretion and transport (Supplementary Data Sheet 3).

Next, we performed qRT-PCR of a subset of genes analyzed for the HTT-AAV groups in the BACHD 2 months group (Supplementary Image 2). Among *Cart*, *Tacr3*, *Hcrt*, *Vip*, *Th*, and *Ghrh*, only *Tacr3* was significantly downregulated compared to the age-matched WT controls.

## Discussion

Using Affymetrix microarray profiling for large-scale gene expression analysis, we assessed changes in hypothalamic transcriptome profiles in mice with overexpression of wtHTT or mHTT in the hypothalamus and the full-length mHTT-expressing transgenic BACHD model. These HD mouse models share a similar metabolic phenotype: an increased food intake and body weight (Hult et al., 2011; Dickson et al., 2022). Understanding hypothalamic changes in HD is relevant as the hypothalamus controls whole body homeostasis through a vast central-peripheral network, and both clinical HD and animal models manifest non-motor symptoms and signs, including metabolic alterations [reviewed in van der Burg et al. (2009) and Cheong et al. (2019)]. We previously showed that overexpression of mHTT (79Q) in the hypothalamus leads to the development of an obese phenotype with leptin resistance. In this study, we show that mHTT (79Q) overexpression in the hypothalamus elicits a more pronounced effect on the local loss of neuropeptides compared to wtHTT (18Q) overexpression, a property of mHTT that is in line with previous work (Hult et al., 2011; Baldo et al., 2013; Dickson et al., 2022). Key hypothalamic populations, including genes involved in catecholamine synthesis and feeding responses, were significantly affected by mHTT 79Q overexpression. Higher levels of *Hdc* mRNA have been reported in HD patients (van Wamelen et al., 2011). In contrast, we report the downregulation of *Hdc* in the overexpression model. Notably, such discrepancies in the histamine systems have also been seen in the study of narcolepsy between patients and animal models (Shan et al., 2015). Hypothalamic neuron populations are versatile in function and communication networks, such as *Hcrt* and *Hdc*, known to regulate both the sleep/wake cycle and feeding (Itowi et al., 1988; Sakai et al., 1995; Jorgensen et al., 2006; Panula and Nuutinen, 2013; Shan et al., 2015). Taken together, more work is needed to map the deleterious route of mHTT in hypothalamic networks and to assess its linkage to non-motor phenotypes. In addition, we highlight the difference between wtHTT and mHTT despite the AAV-mediated dose-increase of HTT protein in both groups, encouraging future studies of a transcriptional link to disease features.

Genes and pathways related to inflammation were prominent across analyses in the AAV datasets and indicated by GSEA in BACHD at 10 months of age. Even though AAV vectors elicit a minimal immunogenic response, high doses of AAV capsid proteins could activate inflammatory pathways, particularly at 4 weeks post-injection, which is an early stage of the transgene expression (Liu and Muruve, 2003). Therefore, an early prominent hypothalamic inflammatory response could be a consequence of viral vector expression rather than HTT-related effects. Future transcriptome analysis with a viral vector control group [i.e., AAV vectors expressing green fluorescent protein (GFP)] is required to discriminate between HTT and viral vector-related inflammatory response. However, notably, in a prior study, we showed that long-term expression of GFP using AAV vectors in mice did not change body weight (Hult et al., 2011). Differential expression analysis of 79Q vs. 18Q revealed no genes passing an adj. *p* < 0.05, however, GSEA indicated suppression of immune-related pathways in the top-ranked outputs. In contrast to the AAV vector model focusing on dose increase selectively in the hypothalamus, BACHD mice express a transgenic full-length mHTT throughout the whole body. This enables us to study how the regional variations in mHTT expression and the extent of disturbed brain-body crosstalk modify disease features. Despite the shared features of increased weight gain and food intake in the two models, there are differences in metabolic profiles (Gray et al., 2008; Hult et al., 2011). Microarray analysis of the hypothalamus in both BACHD groups found genes with a lower magnitude of expression and all adj. *p* > 0.05. Notably, no pathways identified by GSEA-KEGG were shared between the early- and late stage of disease in BACHD compared to their respective age-matched WT littermates. GSEA indicated that gonadotropins and luteinizing hormone signaling pathways might be affected toward the late stage of the disease (10 months). Among leading-edge genes was *Cga*, which encodes the alpha subunit of glycoprotein hormones (in humans: chorionic gonadotropin, luteinizing hormone, follicle-stimulating hormone, and thyroid stimulating hormone) (Bellisario et al., 1973; Querat, 2021). Furthermore, a significant enrichment of inflammatory pathways was only present in 10 months old BACHD vs. WT, where the inflammatory genes such as *H2-Aa* and *Cd74* had comparable expression levels in 2 months old BACHD to WT littermates. Taken together, we can conclude that there is an induction of inflammatory response in the hypothalamus of the AAV model and BACHD model of HD. However, due to the limitations of using only transcriptomics in the present study, interpretation of a pro- versus anti-inflammatory state needs to be conducted in future studies. Hypothalamic inflammation can be caused by obesity and dictated by residential microglia by integrating dietary and hormonal signals from the periphery (Gao et al., 2014; Valdearcos et al., 2014; Dorfman and Thaler, 2015; Jais and Brüning, 2017), notable for the BACHD model and their hyperphagic obesity phenotype (Gray et al., 2008). Expressing mHTT in peripheral tissues can also contribute to metabolic alterations (i.e., adipose tissue) and modify susceptibility to obesity (Lee and Shin, 2017). Furthermore, previous hypothalamic transcriptome analysis of diabetic obese mice with insulin and leptin resistance showed that the top overrepresented pathways included “oxidative phosphorylation” (Gao et al., 2012). Similarly, GSEA-KEGG datasets showed that the “Oxidative phosphorylation” pathway was in the leading-edge gene set in BACHD 10 months old vs. WT, while log2(FC) was lower at 2 months old BACHD vs. WT group. These findings suggest alterations in the “Oxidative phosphorylation” pathway in 10 months old BACHD mice might be a consequence of the BACHD metabolic disturbances, which are prominent at 10 months of age (Gray et al., 2008). In line with this, strikingly, none of the pathways were shared between 2 months and 10 months old BACHD datasets. The obese phenotype of BACHD mice could be a confounding factor influencing the hypothalamic transcriptome profile. However, one of the disease hallmarks is the biphasic changes in both tissue-specific transcriptional dysregulation and movement symptoms at different HD stages (Kirkwood et al., 2001; Chen et al., 2013). As HD progresses, a significant shift from one direction to another is well-known to occur in DA transmission and corticostriatal glutamate transmission (Cepeda et al., 2007; Laprairie et al., 2015). Therefore, the HD biphasic and time-dependent changes could also cause the non-linear transcriptome profile of 2–10 months-old BACHD hypothalami.

Alterations of sterol- and cholesterol metabolism that provide the main components of the myelin sheath and precursors of steroid hormones (Zhang and Liu, 2015) have been found in several brain areas of HD, including the striatum (Sipione et al., 2002; Valenza et al., 2005; Block et al., 2010). In both 18Q vs. WT and 79Q vs. WT, we found a significant impact on genes and pathways related to terpenoid, sterol, and cholesterol metabolism. *Stoml3* and *Sv2c* associated with dopaminergic signal transmission were among the top 10 differentially downregulated in 79Q vs. WT (Janz and Südhof, 1999; Dardou et al., 2011; Schmitt et al., 2016; Dunn et al., 2017). DAVID Functional Annotation Clustering of genes with adj. *p* < 0.05 found a gene set of 37 genes that were shared between 18Q vs. WT and 79Q vs. WT. Loss of cholesterol and FA in the hypothalamus would have a critical impact on several biosynthetic processes of the neuroendocrine system. Further, disruption of neuronal signaling, autophagy, and insulin resistance that have been reported in HD (Lalic et al., 2008; Milnerwood and Raymond, 2010; Martin et al., 2015; Blumenstock and Dudanova, 2020), can be caused by cholesterol depletion in neurons (Fukui et al., 2015).

## Conclusion

We show that significant transcriptional changes in the hypothalamus can be induced by both wtHTT and mHTT overexpression. Sterol- and cholesterol metabolism alterations found in other brain areas of HD can be induced in the hypothalamus by selective overexpression of wtHTT 18Q and mHTT 79Q fragments. We further show that overexpression of mHTT causes pronounced downregulation in catecholamine- and other hypothalamic populations that could have functional implications for the early body weight gain and food intake observed in mHTT 79Q mice. Lastly, the ubiquitous expression of full-length mHTT in BACHD mice causes a milder effect on the hypothalamic transcriptome. Taken together, this further verifies the hypothalamus, with its extensive communications in the brain and periphery, as a candidate area to consider in diseases with ubiquitous expression of the mutant protein, as in HD. Further studies are warranted to validate the biological roles of processes and pathways reported here and assess the significance of disease features in each HD model.

## Data availability statement

The data discussed in this publication have been deposited in NCBI’s Gene Expression Omnibus (Edgar et al., 2002) and are accessible through GEO Series accession number GSE215217.

## Ethics statement

This study was reviewed and approved by Lund University Animal Welfare and Ethics committee in the Lund-Malmö region (Ethical permit numbers: 12585/2017, M20-11, M65-13, and M135-14).

## Author contributions

RS-K, ÅP, ED, and MB designed the experiments from RS-K and ÅP. ED, AD, RS-K, and SL performed the experiments. ED, NA, AD, and RS-K analyzed the data. ED, RS-K, and AD wrote the first draft of the manuscript. All authors reviewed the manuscript and approved the final version.

## Footnotes

1
https://cran.r-project.org/web/packages/pheatmap/index.html

2
https://david.ncifcrf.gov/

3
https://www.kegg.jp/

4
https://www.genome.jp/kegg/

## Supplementary material

Supplementary Image 1 Gene set enrichment analysis (GSEA) with a leading-edge analysis of KEGG pathways in the bacterial artificial chromosome (BAC)-mediated transgenic mouse model (BACHD) datasets. In the limma of microarray datasets, none of the genes in BACHD datasets passed adj. *p*-value < 0.05. GSEA of KEGG pathways was performed. **(A)** GSEA-KEGG of the BACHD vs. WT (2 months of age) dataset. **(B)** BACHD vs. WT (10 months of age). For NES > 0, 13 KEGG pathways were identified by GSEA, of which the top 10 are shown here. One KEGG pathway, “Oxidative phosphorylation” was found for NES < 0 in BACHD vs. WT (10 months of age). Limma, linear models for microarray data, NES, normalized enrichment score, WT, wild type.

Supplementary Image 2 Quantitative real-time polymerase chain reaction (qRT-PCR) of hypothalamic genes in bacterial artificial chromosome (BAC)-mediated transgenic mouse model (BACHD) mice at 2 months of age. Gene expression analysis was performed with hypothalamic RNA samples from BACHD mice at 2 months of age (early stage of disease progression) and age-matched wild type (WT) controls. **(A)** Cocaine and amphetamine-regulated transcript (*Cart*), **(B)** Tachykinin receptor 3 (*Tacr3, p* = 0.0173), **(C)** Hypocretin neuropeptide precursor *(Hcrt)*, **(D)** Vasoactive intestinal peptide (*Vip*), **(E)** Tyrosine hydroxylase (*Th*), and **(F)** Growth hormone-releasing hormone (*Ghrh*). Data are expressed as mRNA expression relative to the mean of age-matched WT controls. The data are represented as scatter dot plots, bars represent mean ± SEM, and a two-tailed Mann-Whitney test was used to analyze the data.

Supplementary Table 1 The list of primer sequences used for qRT-PCR analysis.

Supplementary Data Sheet 1 The limma analysis datasets of 18Q vs. WT, 79Q vs. WT, 79Q vs. 18Q, BACHD vs. WT 2 months of age, and BACHD vs. WT 10 months of age. In addition, supplementary data from Figure 1, the 79Q vs. 18Q and BACHD groups’ volcano plots, where no gene passed adj. *p*-value < 0.05 in the limma, a list for the top genes filtered on only log2(FC) and log2(FC) > 1 or log2(FC) < −1 is provided.

Supplementary Data Sheet 2 DAVID Functional Annotation Analyses in 18Q vs. WT and 79Q vs. WT.

Supplementary Data Sheet 3 Gene Set Enrichment Analysis (GSEA) of gene ontology (GO).

Supplementary Data Sheet 4 Gene Set Enrichment Analysis (GSEA) of KEGG pathways.

## Abbreviations

AAV: adeno-associated viruses
BAC: bacterial artificial chromosome
BACHD: bacterial artificial chromosome (BAC)-mediated transgenic mouse model
BMI: body mass index
BP: biological process
*Cart*: cocaine- and amphetamine-regulated transcript
*Cxcl*: C-X -C motif chemokines
DAVID: database for annotation visualization and integrated discovery
*Ddc*: dopa decarboxylase
ES: enrichment score
FA: fatty acid
FC: fold change
GAPDH: glyceraldehyde 3-phosphate dehydrogenase
GO: gene ontology
GFP: green fluorescent protein
*Ghrh*: growth hormone-releasing hormone
*Gnrh*: gonadotropin-releasing hormone
GSEA: gene set enrichment analysis
*Hcrt*: orexin/hypocretin protein
HD: Huntington’s disease
*Hdc*: histidine decarboxylase
*HTT*: huntingtin
*Idi1*: isopentenyl-diphosphate delta isomerase 1
Limma: linear models for microarray analysis
*Ldlr*: low-density lipoprotein receptor
*Mfsd2a*: major facilitator superfamily domain-containing 2A
NES: normalized enrichment score
*Nm*: neuromedin
Oprm1: opioid receptor mu 1
PCA: principal component analysis
qRT-PCR: quantitative real-time polymerase chain reaction
rAAV5: recombinant AAV vector serotype 5
RIN: RNA integrity number
RMA: robust multi-array average
*Slc18a2*: the gene encoding the vesicular monoamine transporter 2 (VMAT)
*Stoml3*: stomatin-like protein 3
Syn-1: synapsin-1
*Sv2c*: synaptic vesicle glycoprotein 2C
*Tacr3*: tachykinin receptor 3
*Th*: tyrosine-hydroxylase
*Vip*: vasoactive intestinal peptide
WT: wild-type
wtHTT: wild-type huntingtin
mHTT: mutant huntingtin.
