# Supplementary figures and images for: Microarray profiling of hypothalamic gene expression changes in Huntington’s disease mouse models
Source: Front Neurosci. 2022 Nov 3;16:1027269. doi: 10.3389/fnins.2022.1027269 (PMC9671106; doi:10.3389/fnins.2022.1027269)

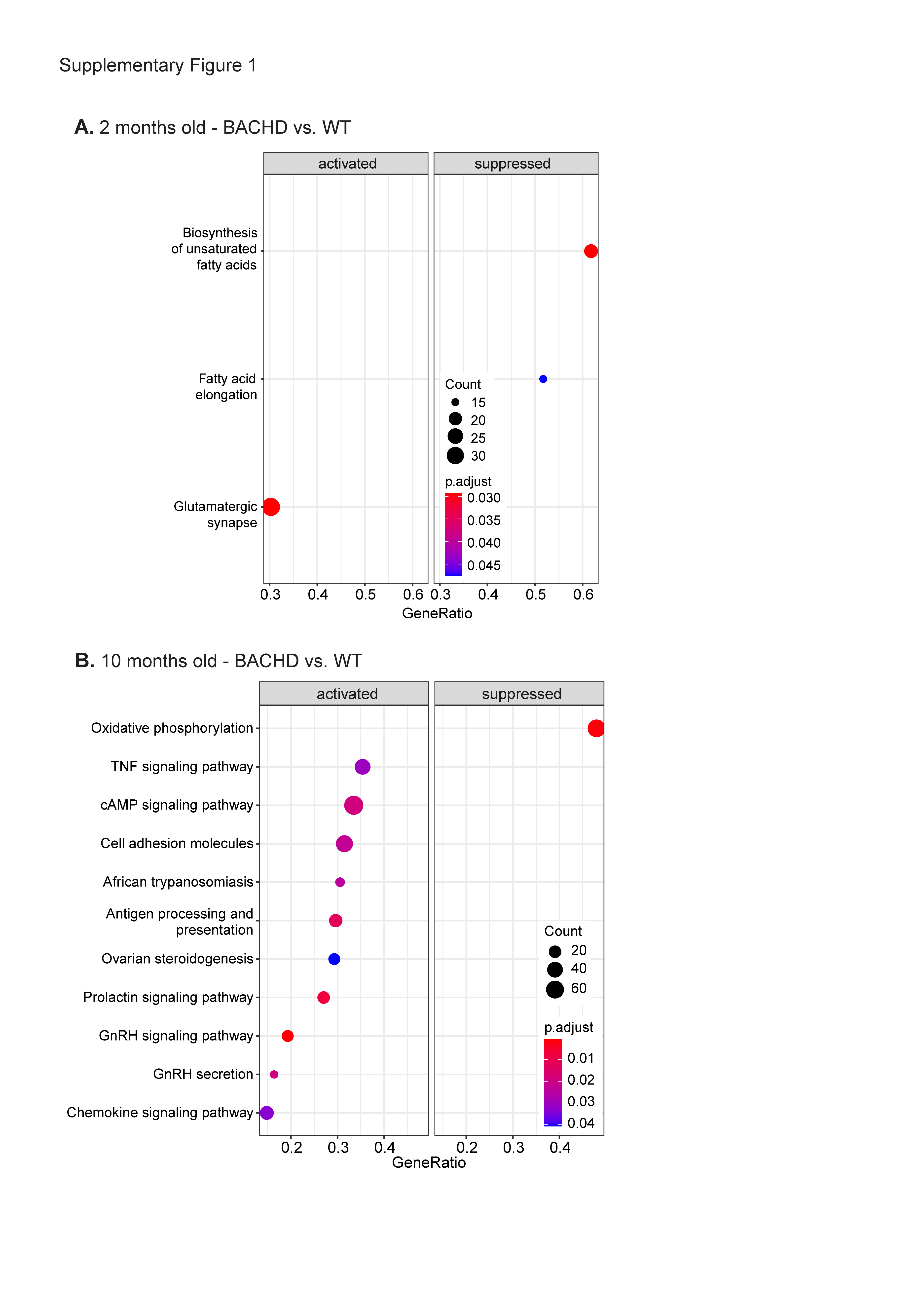

Supplement: Supplementary Image 1 — Gene set enrichment analysis (GSEA) with a leading-edge analysis of KEGG pathways in the bacterial artificial chromosome (BAC)-mediated transgenic mouse model (BACHD) datasets. In the limma of microarray datasets, none of the genes in BACHD datasets passed adj. p-value < 0.05. GSEA of KEGG pathways was performed. (A) GSEA-KEGG of the BACHD vs. WT (2 months of age) dataset. (B) BACHD vs. WT (10 months of age). For NES > 0, 13 KEGG pathways were identified by GSEA, of which the top 10 are shown here. One KEGG pathway, “Oxidative phosphorylation” was found for NES < 0 in BACHD vs. WT (10 months of age). Limma, linear models for microarray data, NES, normalized enrichment score, WT, wild type. [file Image_1.tif]

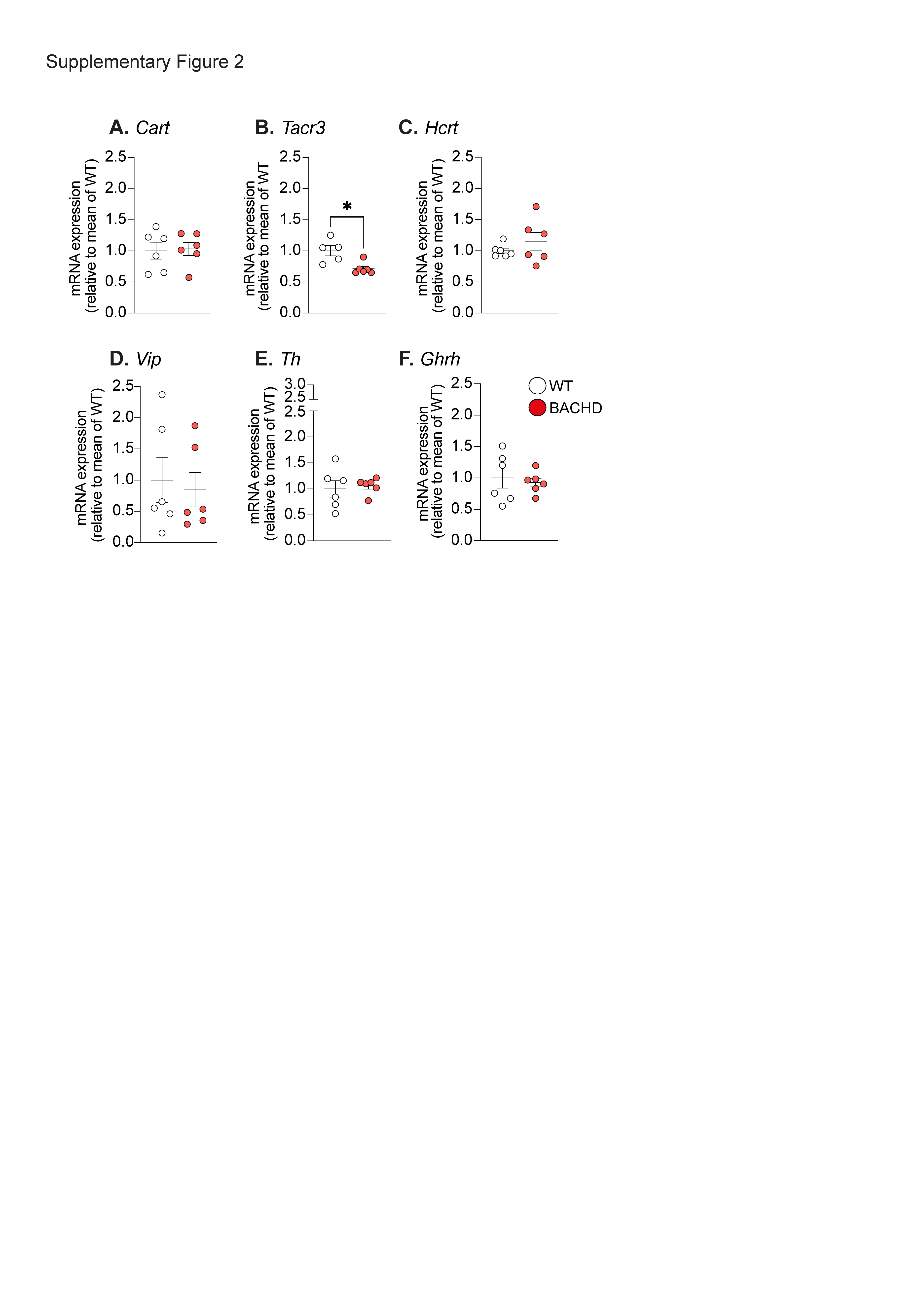

Supplement: Supplementary Image 2 — Quantitative real-time polymerase chain reaction (qRT-PCR) of hypothalamic genes in bacterial artificial chromosome (BAC)-mediated transgenic mouse model (BACHD) mice at 2 months of age. Gene expression analysis was performed with hypothalamic RNA samples from BACHD mice at 2 months of age (early stage of disease progression) and age-matched wild type (WT) controls. (A) Cocaine and amphetamine-regulated transcript (Cart), (B) Tachykinin receptor 3 (Tacr3, p = 0.0173), (C) Hypocretin neuropeptide precursor (Hcrt), (D) Vasoactive intestinal peptide (Vip), (E) Tyrosine hydroxylase (Th), and (F) Growth hormone-releasing hormone (Ghrh). Data are expressed as mRNA expression relative to the mean of age-matched WT controls. The data are represented as scatter dot plots, bars represent mean ± SEM, and a two-tailed Mann-Whitney test was used to analyze the data. [file Image_2.tif]
