# Supplementary material for: Microarray profiling of hypothalamic gene expression changes in Huntington’s disease mouse models
Source: Front Neurosci. 2022 Nov 3;16:1027269. doi: 10.3389/fnins.2022.1027269 (PMC9671106; doi:10.3389/fnins.2022.1027269)
Supplement: Supplementary Table 1 — The list of primer sequences used for qRT-PCR analysis. [file Table_1.DOCX]

## Supplementary Table 1

| Gene name | Direction | Sequence (5’🡪3’) |
| --- | --- | --- |
| Cart | F | GTTGCAGATCGAAGCGTTG |
|  | R | ATCGGAATGCGTTTACTCTTGA |
|  |  |  |
| Hcrt | F | CCATCTTCTATCCTTGTC |
|  | R | GTCTTTATTGCCATTTACC |
|  |  |  |
| Tacr3 | F | GTCACCTACACCATCGTT |
|  | R | GTCACCACCACAATATCATC |
|  |  |  |
| Hdc | F | GAGAAGGCTGGCTTGATT |
|  | R | TCGGAGTGAGAAGTTGTC |
|  |  |  |
| Th | F | TTCTCAACCTGCTCTTCT |
|  | R | TGGCTTCAAATGTCTCAAA |
|  |  |  |
| Vmat2 | F | GGCTATCCACATTCTTCAGA |
|  | R | ACACCAGACTTCACATCA |
|  |  |  |
| Β-actin | F | GCTGTGCTATGTTGCTCTA |
|  | R | TCGTTGCCAATAGTGATGA |
| Gapdh | F | AACCTGCCAAGTATGATG |
|  | R | GGAGTTGCTGTTGAA |
|  |  |  |
| Ddc [1] | F | GCAGTGCCTTTATCTGTCCT |
|  | R | GAATCCTGAGTCCTGGTGAC |
|  |  |  |
| Vip | F | GATAGGCTGCTGTGTTAC |
|  | R | AGAGATGAATCCGTGAGA |
|  |  |  |
| Ghrh | F | ATCTTCACCACCAACTAC |
|  | R | ATGTCCTGGATCACTTTC |

References

1. Vogelgesang, S., et al., *Analysis of the Serotonergic System in a Mouse Model of Rett Syndrome Reveals Unusual Upregulation of Serotonin Receptor 5b.* Frontiers in Molecular Neuroscience, 2017. **10**.
